# Prevalence of Dermatological Presentations of Canine Leishmaniasis in a Nonendemic Area: A Retrospective Study of 100 Dogs

**DOI:** 10.1155/2014/374613

**Published:** 2014-02-05

**Authors:** Roberta Perego, Daniela Proverbio, Giada Bagnagatti De Giorgi, Eva Spada

**Affiliations:** Dipartimento di Scienze Animali Per la Salute, la Produzione Animale e la Sicurezza Alimentare, University of Milan, Via Celoria 10, 20133 Milan, Italy

## Abstract

This retrospective study determined the prevalence of dermatological lesions associated with canine leishmaniasis (CanL) in a nonendemic area in Italy. The medical records of 131 dogs with CanL were reviewed and, of these, 115/131 dogs (88%) had dermatological manifestations of which 100/131 dogs (76%) met the inclusion criteria. Sixty-two percent of dogs were male and 38% were female and the mean age was 6.4 years. Thirty-two percent of dogs were mixed breeds; the remainder represented a variety of pure breeds. In 79% of dogs dermatological signs occurred in association with systemic signs of CanL, whilst 21% of dogs had only dermatological manifestations. The most common dermatological manifestation was exfoliative dermatitis (74%), followed by ulcerative (18%) and nodular (11%) lesions. In 51% of dogs the lesions were localized mainly on the pinnae, head, and pressure points; in the remaining 49% lesions were generalized. The only statistically significant association was between Retriever breed and animals with only dermatological signs (*P* = 0.0034, OD 5.97, CI 0.996–37.933). In this study dermatological manifestations of CanL were very commonly reported, and their prevalence is similar to previous studies in endemic areas despite the fact that dogs living in nonendemic areas are not exposed to repeated infectious bites and continuous stimulation of the dermal immune system.

## 1. Introduction

Canine leishmaniasis (CanL) is a zoonotic chronic wasting disease that is endemic along the Mediterranean basin, subtropical and tropical regions of Asia, South and Central America [1], and more recently also in the United States of America [2]. CanL is caused by the intracellular protozoan of the genus *Leishmania *[3], and, in the Old World, the parasite is transmitted to man and animals by blood-sucking sandflies of the genus *Phlebotomus* [3]. Clinical features associated with CanL are variable and often nonspecific [4–6], and the broad spectrum of clinical manifestations results from several pathogenic mechanisms that characterize the disease and the immune responses of different hosts [3]. Dermatological manifestations are the most commonly reported clinical features of CanL [5, 7–9]. Ferrer et al. [10], in a field study of 43 naturally infected dogs, first identified four different dermatological pictures in dogs with CanL: alopecia and desquamation, ulcerative dermatosis, nodular disease, and pustular dermatitis (considered in subsequent studies to be the most common cutaneous manifestations of this disease). In studies in endemic areas the most commonly reported skin lesions are exfoliative dermatitis with generalized or focal alopecia/hypotrichosis and cutaneous or mucosal ulcerations, followed much less frequently by nodular dermatitis and sterile pustular dermatitis [4, 5, 8, 9, 11, 12].

Other manifestations often reported in cases of CanL are onychogryphosis, paronychia, nasal and digital depigmentation, and hyperkeratosis [5, 8, 13–15].

Additionally, there are a number of atypical cutaneous forms of CanL such as papular dermatitis [16] and inoculation chancre, which are more frequently localised on the less furred areas of the head [9, 14].

In human leishmaniasis it is proposed that the efficiency of the immune response may account for the variety of dermatological lesions [17, 18]. The same may be true in dogs where the exfoliative dermatitis form has been associated with an effective local immune response, manifested by activation of epidermal Langerhans cells, upregulation of MHC-II molecules on keratinocytes, dermal infiltration by CD8+ and a reduced number of CD4+ cells, presence of CD21+ cells, and a relatively low parasite burden [19, 20]; whereas the nodular form is usually characterized by low epidermal expression of MHC-II molecules, small numbers of T cells in the dermal infiltrate, and a high parasite burden, indicating increased susceptibility of the host [5, 13, 19] although exactly the opposite has also been suggested [15]. There is also evidence that the interaction between Leishmania spp. and the immune system in the skin is influenced by the repeated infectious bites and the simultaneous intradermal injection of sandfly saliva [12].

Despite the extensive literature concerning CanL, few (and no recent) studies have focused exclusively on the prevalence and description of the cutaneous lesions [8, 10]. Most data are derived from epidemiological studies performed exclusively on dog populations in endemic areas [4, 5, 9], with the sole exception of the study by Slappendel [13], carried out in the Netherlands. To the authors' knowledge there are no studies that analyze the prevalence of cutaneous features of CanL in dogs from a nonendemic area.

The aim of this retrospective study was to determine the prevalence and type of dermatological lesions in a large group of dogs with CanL referred to the Veterinary Teaching Hospital in Milan, Italy, which is in a currently nonendemic area for CanL [21]. The authors hypothesized that because the response of the dermal immune system is known to be influenced by repeated infectious bites, dermatological features in dogs from nonendemic areas may be different to those described in endemic areas. The association between some parameters of signalment and the presence of cutaneous signs was also evaluated.

## 2. Materials and Methods

### 2.1. Case Selection

A retrospective study was performed. The medical records of all cases of confirmed leishmaniasis in dogs presented at the Teaching Hospital of the University of Veterinary Medicine in Milan between 2000 and 2013 were reviewed. Dogs were eligible for inclusion in the study if they fulfilled the following criteria.

1. A complete physical examination at the first consultation with a detailed description of signs referable to CanL as previously reported in the literature [22].
2. Living in nonendemic area of Milan.
3. Presence of at least one common or atypical dermatological manifestation referable to CanL according to the literature [22].
4. A diagnosis of leishmaniasis established by clinicopathologic abnormalities, positive serology for *Leishmania infantum* using IFA and cytologic identification of leishmania amastigotes, or detection of parasite DNA using polymerase chain reaction in either lymph node or bone marrow aspirates according to diagnostic criteria previously described [22], and finally, in cases with nodular presentation, direct observation of the parasites by skin biopsies as described previously [22].

All dogs with other neoplastic, inflammatory, endocrine, immunologic, and genetic diseases potentially associated with dermatological signs were excluded. Furthermore dogs were also excluded if they had concomitant infectious diseases (e.g., babesiosis, ehrlichiosis, dirofilariasis), diagnosed by parasitological or serological examinations, or both.

### 2.2. Medical Records Review

Information extracted from the medical records of each dog with leishmaniasis included in the study comprised signalment (age, sex, breed, provenance/travel history), dermatological signs classified as localized or generalized and as common form (exfoliative dermatitis with or without alopecia, ulcerative dermatitis, nodular dermatitis, pustular dermatitis), common adnexal manifestations (onychogryphosis, paronychia, and nasal/digital hyperkeratosis), or atypical form (papular dermatitis and inoculation chancre) as described in the literature [10, 15] and systemic signs referable to CanL [22].

### 2.3. Statistical Analysis

All statistical analyses were performed using commercial statistical software (MedCalc, v. 12.3.0). For each analyzed parameter the distribution of data recorded was assessed using descriptive statistics. A chi-square test or a Fisher exact test, depending of the number of observations, was used to test for associations between breed, age, sex, and common or atypical dermatological forms. Odds ratio (OR) was used to measure the degree of associations and 95% confidence intervals (CIs) were reported. Significance was based on *P* < 0.05 for all tests.

## 3. Results and Discussion

The medical records of 131 dogs with leishmaniasis were reviewed, and, of these, 16 dogs were excluded because of the absence of dermatological signs at the first consultation. Of the remaining 115/131 dogs (88%) who had CanL with dermatological manifestations, 5 dogs were excluded because of incomplete medical records, 6 dogs because of concomitant infectious diseases, and 4 dogs for other diseases potentially associated with dermatological signs. A total of 100/131 dogs (76%) met the criteria for inclusion in the study.

Of the 100 cases enrolled, 62 dogs (62%) were male (5 castrated) and 38 (38%) were female (12 spayed) and the mean age was 6.4 years (range from 1 to 14 years). Thirty-two dogs (32%) were mixed breeds, 10 (10%) Boxers, 8 (8%) Setters, 7 (7%) Retrievers, 5 (5%) German Shepherds, 5 (5%) Hounds, 4 (4%) Spaniels, 4 (4%) Nordics, 4 (4%) Great Danes, and 3 (3%) Bulldogs, and the remaining 17 dogs (17%) were pure breeds representing 13 different breeds. Thirty-five dogs (35%) came from kennels, 31 dogs (31%) came from private litters, 23 dogs (23%) came from national breeders, and 11 dogs (11%) came from pet stores. All dogs lived in Milan but had previously travelled to areas endemic for CanL.

On the basis of the presence or absence of systemic signs referable to CanL detected on clinical examination the dogs were divided into two groups: seventy-nine dogs (79%) had dermatological and systemic signs of CanL, while 21 dogs (21%) had only dermatological manifestation of CanL and no identifiable systemic signs on clinical examination.

Table 1 reports the distribution of dermatological manifestation in all 100 dogs with CanL. Each dog could have multiple concurrent dermatological manifestations. Specifically, 22 dogs (22%) had more than one dermatological manifestation at the time of first consultation. In 51 dogs (51%) the lesions were localized to one or two body areas; in the remaining 49 dogs (49%) lesions were generalized. Of the 51 dogs with localized cutaneous manifestations, localized exfoliative and/or alopecic lesions were mainly on pinnae (15/51 dogs, 29%), periocular area (11/51 dogs, 22%), muzzle (6/51 dogs, 12%), and elbows (3/51 dogs, 6%); localized skin ulcerations were located over the pressure points (12/51 dogs, 24%) and pinnae (6/51 dogs, 12%). The nodules were present on the head (5/51 dogs, 10%), limbs (3/51 dogs, 6%), and pinnae (3/51 dogs, 6%), varying from 1 cm to 5 cm in diameter. The distribution of dermatological lesions in the group of dogs without systemic signs and only a dermatological manifestation of CanL are reported in Table 1. Each dog could have multiple concurrent dermatological manifestations.

There were no statistically significant associations between sex and age and dermatological manifestations (data not reported). The only statistically significant association was between Retriever breed and the group of patients with only dermatological signs (*P* = 0.0034, OD 5.97, CI 0.996–37.933).

To the authors' knowledge this is the first retrospective study reporting on dermatological presentations of CanL in an area of northern Italy where CanL is not endemic. Results of this study suggest that dermatological manifestations commonly reported in dogs with CanL from endemic areas occur with the same frequency in dogs living in nonendemic areas. In this study 88% of confirmed cases of canine leishmaniasis at the Veterinary Teaching Hospital of the University of Milan over a 13-year period had dermatological manifestations. Our findings support those in the literature that report skin abnormalities in approximately 80%–90% of CanL cases in studies in endemic areas [5, 13, 22].

Also of note, in 21% of these cases only dermatological signs were reported at the first consultation, with no detectable systemic abnormalities. This percentage, similar to that of Cabassu et al. [23] who reported 16.5% of their 104 cases with only skin lesions, emphasizes the importance of careful evaluation of dermatological signs in this disease.

Some breeds such as Boxers, Setters, Retrievers, and German Shepherds appear to be overrepresented, and male dogs seem to be more numerous in our study. This is in partial agreement with the literature which suggests that Boxer, German Shepherd dogs [22], and male dogs [4, 10, 13] are more susceptible to the development of CanL but these high breed prevalences may still reflect the authors' general hospital population.

The prevalence of the different dermatological forms identified in our study, both on the total sample of 100 dogs and on the subgroup of 21 dogs with only skin manifestations, was very similar to those seen in previous studies in endemic areas [4, 5, 9, 10]. In particular, exfoliative dermatitis was the most common. However, in our study we found lower rates of the ulcerative form [4, 5, 8] and onychogryphosis [5, 8] (and conversely a higher prevalence of the nodular form) than reported in previous studies conducted in endemic areas [4, 5, 8]. The real prognosis in the nodular form of Canl is unknown. There are studies that suggest that this form is associated with greater susceptibility to infection [20] but others suggest favourable outcomes [15]. Some authors [12] speculate that such confusion may stem from the fact that some animals with nodular sites of inoculation, a form associated with resistance to disease, are misclassified as having nodular disease and hence have apparently unexpected favourable outcomes.

In our study many of the nodular lesions were localized to the head and pinnae, typical sites of inoculation. The dogs in this study, after visiting an endemic area where it is likely they contracted the infection, lived in a nonendemic area and were no longer subject to continuous stimulation of the skin immune system from repeated infectious bites and the simultaneous intradermal injection of sandfly saliva [12].

Exfoliative dermatitis was usually located on the pinnae, the periocular area, and head, while skin ulcerations were mostly located on pressure points and pinnae, as previously reported in the literature [5, 8, 10].

It is important to note that 22% of the dogs in this study had more than one dermatological form concurrently highlighting once again the extreme variability of the signs of this disease and the difficulty in accurately describing a dermatological form, as already reported by Koutinas et al. in endemic areas of Greece [8] who identified concurrent multiple dermatological forms in approximately 86% of animals in his study.

It should be emphasized that the Retriever breed appears statistically over represented (*P* = 0.0043) in the group of animals with no systemic signs but is important to underline that the small sample size in this subgroup limited our ability to perform statistical analyses and the conclusions may be affected by type II error.

## 4. Conclusions

In conclusion, the results of this study conducted in a nonendemic leishmania area confirm that dermatological manifestations, particularly exfoliative dermatitis, are very common in CanL and are often associated with variable systemic signs. Our results also underline the extreme variability of dermatological lesions in CanL that can easily be confused with other similar conditions.

Our study does not confirm our initial hypothesis that a lower antigenic stimulation associated with the absence of repeated infectious bites would lead to substantial differences in the prevalence and dermatological manifestations between endemic and nonendemic areas. In fact the prevalence and the clinical dermatological forms in our study are quite similar to previous epidemiological studies conducted in endemic areas, despite the lack of continuous antigenic stimulation. The author's data also demonstrates no correlation between sex and age and common or atypical dermatological forms of CanL in a nonendemic area.

## Figures and Tables

**Table 1:** Distribution of dermatological manifestation of CanL in all 100 dogs and in 21 dogs in the group with only dermatological manifestations of CanL and no identifiable systemic signs.

	Number of subjects	Prevalence
Dermatological form or manifestation in 100 dogs		
Exfoliative dermatitis with or without alopecia	74	74%
Ulcerative dermatitis	18	18%
Nodular form	11	11%
Onychogryphosis	10	10%
Nasal and digital hyperkeratosis	9	9%
Nasal depigmentation	1	1%
Sterile pustular dermatitis	1	1%
Dermatological form or manifestation in 21 dogs with only dermatological manifestations		
Exfoliative dermatitis with or without alopecia	13	62%
Ulcerative dermatitis	5	24%
Nodular form	2	10%
Onychogryphosis	1	5%
Nasal and digital hyperkeratosis	2	10%
